# Alterations in the Notch4 pathway in cerebral endothelial cells by the HIV aspartyl protease inhibitor, nelfinavir

**DOI:** 10.1186/1471-2202-9-27

**Published:** 2008-02-26

**Authors:** Aline Grigorian, Rosemary Hurford, Ying Chao, Christina Patrick, T Dianne Langford

**Affiliations:** 1 Department of Pathology, University of California San Diego, La Jolla, USA; 2 Department of Neurosciences, University of California San Diego, La Jolla, USA; 3 Department of Neuroscience, Temple University School of Medicine,, Philadelphia, USA

## Abstract

**Background:**

Aspartyl protease inhibitors (PIs) used to treat HIV belong to an important group of drugs that influence significantly endothelial cell functioning and angiogenic capacity, although specific mechanisms are poorly understood. Recently, PIs, particularly Nelfinavir, were reported to disrupt Notch signaling in the HIV-related endothelial cell neoplasm, Kaposi's sarcoma. Given the importance of maintaining proper cerebral endothelial cell signaling at the blood brain barrier during HIV infection, we considered potential signaling pathways such as Notch, that may be vulnerable to dysregulation during exposure to PI-based anti-retroviral regimens. Notch processing by γ-secretase results in cleavage of the notch intracellular domain that travels to the nucleus to regulate expression of genes such as vascular endothelial cell growth factor and NFκB that are critical in endothelial cell functioning. Since, the effects of HIV PIs on γ-secretase substrate pathways in cerebral endothelial cell signaling have not been addressed, we sought to determine the effects of HIV PIs on Notch and amyloid precursor protein.

**Results:**

Exposure to reported physiological levels of Saquinavir, Indinavir, Nelfinavir and Ritonavir, significantly increased reactive oxygen species in cerebral endothelial cells, but had no effect on cell survival. Likewise, PIs decreased Notch 4-protein expression, but had no effect on Notch 1 or amyloid precursor protein expression. On the other hand, only Nelfinavir increased significantly Notch 4 processing, Notch4 intracellular domain nuclear localization and the expression of notch intracellular domain targets NFκB and matrix metalloproteinase 2. Pre-treatment with the antioxidant Vitamin E prevented PI-induced reactive oxygen species generation and partially prevented Nelfinavir-induced changes in both Notch 4 processing, and cellular localization patterns. Moreover, in support of increased expression of pro-angiogenic genes after Nelfinavir treatment, Nelfinavir did not inhibit angiogenic capacity.

**Conclusion:**

Nelfinavir affects Notch 4 processing that results in induction of expression of the pro-angiogenic genes NFκB and matrix metalloproteinase 2 in cerebral endothelial cells.

## Background

As the first line of defense against substances attempting to enter the CNS, cerebral endothelial cells (CEC) are continually exposed to a variety of blood-borne factors including pathogens such as HIV, and/or pharmacological agents used to treat infection. In fact, CEC activation and compromise of the blood brain barrier occurs during HIV infection of the CNS [1-3]. In addition, protease inhibitors (PIs) used to treat HIV belong to an important group of drugs reported to influence significantly angiogenic capacity and endothelial cell functioning [4]. For example, exposure of endothelial cells to some PIs has been shown to increase oxidative stress, induce signaling dysfunction, mitochondrial dysregulation and promote formation of intercellular gaps [5,6]. Although adverse effects on non-cerebral endothelial cells by HIV PIs are well documented, the mechanisms responsible for dysregulation are poorly understood [4]. Recently, these aspartyl PIs, particularly Nelfinavir (NFV), have been implicated in disruption of the Notch pathway in the HIV-related neoplasm, Kaposi's sarcoma [7]. Notch and amyloid precursor protein (APP) are substrates for the aspartyl protease γ-secretase and represent pathways known to support numerous key points in endothelial cell fitness. Therefore, constant exposure of CEC to PIs circulating in the blood stream in the HIV patient likely affects normal CEC signaling pathways [8,9], such as Notch since its signaling is dependent on protease activity to maintain cell fitness [10,11].

Expressed mainly on endothelial cells, Notch 4 is a member of the transmembrane Notch family of receptors [12,13]. Upon binding by its ligand, Delta4, the C-terminal Notch intracellular domain (NICD) is cleaved by γ-secretase and travels to the nucleus where it associates via the CBF-1, Su(H), Lag-1 (CSL) family of DNA-binding proteins to form transcription activator complexes, [10,11] that regulate, among others vascular endothelial cell growth factor (VEGF), NFκB and HES-1 expression [14,15], all of which are crucial for endothelial cell fitness. In addition, recent reports describe the phosphorylation of NICD by GSK3-β with subsequent transcriptional regulation of NICD target genes [16-18].

Thus, given the importance of maintaining proper endothelial cell signaling at the blood brain barrier, we hypothesized that Notch expression and processing may become vulnerable to dysregulation in CEC during exposure to PIs. Our results show that the HIV PI NFV significantly affects Notch 4 expression and processing in a vitamin E-sensitive manner that appears independent from GSK3-β phosphorylation levels. NFV exposure also increases Notch 4 NICD nuclear localization and the expression of NICD target genes NFκB and matrix metalloproteinase 2 (MMP2). In support of increased expression of pro-angiogenic genes after NFV treatment, NFV did not inhibit angiogenic capacity in CEC.

Increased understanding of crosstalk between PIs and CEC is critical to improve treatment, predict complications and manage HIV-associated CNS complications in the HIV patient adherent to a PI-containing anti-retroviral regimen. In particular, understanding alterations in signaling cascades relevant to endothelial cell fitness is of crucial importance during viral rebound when anti-retroviral drugs in the blood are accompanied by circulating HIV-infected immune cells.

## Methods

### Cerebral endothelial cell culture and PI treatments

The effects of the HIV PIs SQV, INV, NFV, and RTV on cell viability and signaling were investigated in human CEC (ScienCell Research Laboratories, San Diego, CA and Cell Systems, Kirkland, WA). CEC were maintained at no greater than 70% confluence in endothelial cell medium (ECM), which includes 5% fetal bovine serum (FBS), 1% endothelial cell growth supplement, and 1% penicillin/streptomycin solution (ScienCell Research Laboratories). Cells were routinely grown in ECM, incubated at 37°C in 5% CO_2_, and the medium was replaced every three days. To slow cell metabolism and bring the cells to a resting state, CEC were pre-incubated in ECM containing 1% FBS overnight prior to experimental treatments. CEC were treated with 5 μM of the PIs SQV, INV, NFV, RTV or 5 μM INV/1 μM RTV (NIH AIDS Research & Reference Reagent Program, Germantown, MD) for varying lengths of time, and untreated cells in 1% FBS were used as control. Studies have shown that plasma levels of PIs in patients taking these drugs are approximately 5 μM [19]. A dose response experiment determined 5 μM PI to be non-lethal to CEC and was therefore used in the time course experiments. PI treatments for 48, 72 and 96 h were refreshed daily by replacing media containing drug to ensure a consistent PI concentration throughout the time course [5]. For LY29004 (Calbiochem, La Jolla, CA) treatment, CEC were exposed to 10 μm inhibitor for 10 min followed by PIs. After treatments, cells were rinsed with PBS and harvested for assays.

### Cell viability assays

Trypan blue exclusion assays were performed to determine the effects of time and drug concentration on cell viability. PI concentrations included 0.5, 1, 5, and 25 μM, in addition to untreated control. Cell viability was also determined at the non-lethal dose of 5 μM PI over time including 24, 48, 72, and 96 h. CEC from each dose and time treatment were rinsed with PBS, detached using a trypsin solution (Invitrogen, Carlsbad, CA), harvested by gentle centrifugation, and resuspended in PBS/trypan blue solution (1:1, vol:vol). Cell viability was determined by counting stained (dead) versus non-stained (live) cells, as previously described [20].

### H_2_DCFDA Staining

H_2_DCFDA (2', 7'-dichlorodihydrofluorescein diacetate) is a cell-permeable reactive oxygen species (ROS) indicator that remains non-fluorescent until removal of acetate groups by intracellular esterases and oxidation occurs within the treated cells. H_2_DCFDA staining is widely used for assessing overall oxidative stress.

CEC were grown on poly-L-lysine-coated thermanox plastic coverslips (Thermo-Fisher, Pittsburgh, PA) and treated for 1 and 48 h with PIs and/or Vitamin E acetate (Sigma-Aldrich Co., St. Louis, MO). A dose response for protection against ROS generation was conducted using 25, 50, 100, 200, and 500 μM Vitamin E. Cells undergoing Vitamin E treatment were pre-treated 1 h prior to PI treatment and Vitamin E remained in the media throughout the drug incubation. CEC were rinsed gently with PBS and stained with 75 μM H_2_DCFDA in PBS for 15 min at 37°C. The stain was rinsed off by gently dipping the coverslips in PBS several times. Coverslips were mounted on glass slides and cells were observed by fluorescence microscopy using the Olympus BX41 microscope (Olympus, Melville, NY). Images were captured for computer analysis using Optronics MagnaFire SP software (Optronics, Goleta, CA).

### Immunocytochemical analyses

For immunocytochemical characterization of Notch 1, Notch 4, APP and CD31 expression, CEC were plated onto poly-L-lysine-coated coverslips for 24 h in ECM containing 1% FBS and fixed for 20 min at room temperature in 4% paraformaldehyde. Paraffin embedded samples of human frontal cortex collected at autopsy from an HIV sero-negative adult with no brain alterations were obtained from the HIV Neurobehavioral Research Center (University of California San Diego, San Diego, CA). CEC and 40 μm vibratome tissue sections were incubated overnight with the mouse monoclonal antibodies against Notch 1 (1:50), Notch 4 (1:50), APP (1:500), NICD (1:500, Santa Cruz Biotechnology, Inc, Santa Cruz, CA), or the endothelial cell marker, CD31 (1:3000, Chemicon International, Temecula, CA). For immunocytochemical analyses, cells and tissue were single or double labeled with the antibodies and detected with either fluorescein isothiocyanate (FITC, Vector Laboratories Inc., Burlingame, CA) and/or the Tyramide Signal Amplification™-Direct (Red) system (NEN Life Sciences, Boston, MA). FITC analysis required 1 h incubation at room temperature with FITC-conjugated horse anti-mouse antibodies (1:100), while Tyramide Red analysis required 1 h incubation with biotinylated anti-mouse or rabbit antibodies (1:100) followed by Streptavidin HRP (1:500 dil) and Tyramide Red (1:50 dil) labeling. Tissue sections and cells were imaged with a Zeiss 63× (N.A. 1.4) objective on an Axiovert 35 microscope (Zeiss, Germany) with an attached MRC1024 laser scanning confocal microscope (LSCM) system (BioRad, Wattford, UK).

For Notch 4 cellular localization, CEC plated onto poly-L-lysine-coated coverslips in ECM containing 1% FBS were untreated or treated with SQV, INV, NFV, RTV, Vitamin E, or Vitamin E/NFV for 48 h, followed by fixation for 20 min at room temperature in 4% paraformaldehyde. Cells were then double labeled with anti-Actin antibody (1:200) (Chemicon, Temecula, CA) followed by incubation with Texas red secondary antibody (1:100, Vector Labs) and anti-NICD antibody (1:100) (Abcam Inc, Cambridge, MA) followed by incubation with FITC secondary (1:100, Vector labs). Cells from at least ten random fields of view and > 200 cells for each condition were analyzed for NICD cytoplasmic and nuclear localization using the Olympus BX41 microscope (Olympus, Melville, NY). Images were captured for computer analysis using Optronics MagnaFire SP software (Optronics, Goleta, CA) and the percentage of cells positive for NICD nuclear localization was determined. Baseline (untreated) levels of detectable nuclear NICD for Notch 4 are relatively low in CEC, thus comparisons were easily assessed.

### Western Analyses

Total protein was isolated from control and treated cells. Cells were washed with ice-cold PBS and solubilized on ice for 45 min using lysis buffer (50 mM Tris Hal, pH 7.4; 5 mM EDTA; 150 mM NaCl; 1% Triton X-100; 0.4% sodium cacodylate; Protease Inhibitor Cocktail III [Calbiochem, San Diego, CA] and Phosphotase Inhibitor Cocktail I [Calbiochem]). Cell lysate was collected by scraping each well and by sonication for 3 sec at low frequency to ensure lysis (Fisher Scientific Model 100 Sonic Dismembrator, Tustin, CA). After a 10 min centrifugation at 12,000 rpm at 4°C, supernatant was collected for Western analysis. Protein concentrations were determined using the BCA Protein Assay kit (Pierce, Rockford, IL) following the manufacturer's protocol. Twenty μg of total protein was loaded per well onto a 4–12% Bis-Tris NuPage Gels (Invitrogen, Carlsbad, CA) and separated by electrophoresis for 1 h at 200 V. Proteins were transferred onto Immobilon-P PVDF membranes (Millipore, Bedford, MA) for 24 h at 10 V. Notch 1 (H-131, 1:500, Santa Cruz Biotechnology, Inc, Santa Cruz, CA), Notch 4 (H-225, 1:1000, Santa Cruz Biotechnology, Inc, Santa Cruz, CA), APP (A4, 1:1000, Chemicon International, Temecula, CA), phosphorylated-GSK3-β (1:2500, Cell Signaling, Beverly, MA), total-GSK3-β (0011-A, 1:2500, Santa Cruz Biotechnology, Inc, Santa Cruz, CA), NFκ-B (1:200, Santa Cruz Biotechnology, Santa Cruz, CA), VEGF (1:1000, R&D Systems), MMP2 (1:400, Abcam, Inc., Cambridge, MA) and HES-1 (1:2500, BD Transduction Laboratories) primary antibodies were used for immunolabeling followed by horseradish peroxidase (HRP) tagged secondary antibodies (1:5000, American Qualex, San Clemente, CA). Enhanced chemiluminescence was detected with the Western Lightning Chemiluminescence Reagent Plus kit (PerkinElmer Life Sciences, Boston MA) and recorded using the BioRad VersaDoc Imaging System Model 3000 (BioRad, Hercules, CA). To calculate levels of NICD in CEC, the percentage of NICD compared to total Notch was determined in cells treated with PIs as previously described (Shawber et al., 2003; Curry et al., 2005). Briefly, the percentage of Notch processed to NICD corresponding to Notch 1 (110 kDa) and 4 (70 kDa) NICD was calculated by subtracting the NICD band densities from the combined densities of full length (Notch 1 207 kDa; Notch 4 220 kDa) plus processed products.

### quantitative RT (qRT) PCR

Total RNA was purified from CEC treated with PIs for 1 and 48 h. Cells were washed with PBS, re-suspended in PBS and nucleic acid was isolated with TRIzol LS (1 ml/10^7 ^cells) and chloroform. After vigorous vortexing, cells were incubated for 10 min, centrifuged at 12,000 × g for 15 min at 4°C, and the top aqueous layer was collected and mixed with 1 μl glycogen and an equal volume of 2-propanol. After 10 min of precipitation on ice, samples were centrifuged at 12,000 × g for 15 min at 4°C and the supernatant was discarded. Pellets were washed with 70% ethanol, air dried for 10 min, resuspended in DEPC water and heated to 60°C for 10 min before quantitation. The StrataScript First-Strand Synthesis System was used to synthesize (Stratagene, La Jolla, CA) cDNA following the manufacturer's protocol. Briefly, 1 μg of total RNA was combined with oligo (dT) primer and incubated at 65°C for 5 min. The reactions were slowly cooled to room temperature to allow the primers to anneal to the RNA and the synthesis reaction was prepared by adding, in order, 10× first-strand buffer, RNase Block Ribonuclease Inhibitor (40 U), 100 mM dNTPs, and StrataScript reverse transcriptase (50 U). After 1 h incubation at 42°C for the cDNA synthesis reaction, the samples were heated to 90°C for 5 min and the RNA was quantified. One μg cDNA/sample was submitted to the Center for AIDS Research Genomics Core at UCSD for qRT PCR of target genes Notch 1, Notch 4, APP, HES-1, MMP2 and NFκB, VEGF and Porphobilinogen deaminase, (PBGD) as the reference gene. Primer sequences of target genes were selected from Universal Probe Library (Roche Diagnostics, Alameda, CA) and purchased from Sigma Proligo (Sigma-Proligo, Boulder, CO). Primer specificity for the target genes was confirmed by BLAST at GenBank: NOTCH1-[GenBank: NM 017617]-left, 5'-TGC TGG AGG ACC TCA TCA ACT-3', right, 5'-CAG TGC AGG GCG GAC TTG-3'; NOTCH4-[GenBank: NM 004557]-left, 5'-GGC GAG GAC AGC ATT GGT-3', right, 5'-CAT CAC AAC TCC ATC CTC ATC AA-3'; APP-[GenBank: NM 000484]-left, 5'-GGA ATC TTT GGA ACA GGA AGC A-3', right, 5'-TCC ACT CTG GCC ATG TGT GT-3'; HES-1-[GenBank: NM 005524]-left, 5'-ATG GAG AAA AAT TCC TCG TCC C-3', right, 5'-TTC AGA GCA TCC AAA ATC AGT GT-3'; MMP2-[GenBank: NM 004530]-left, 5'-CCG TCG CCC ATC ATC AAG TT-3', right, 5'-CTG TCT GGG GCA GTC CAA AG-3'; NFκB-[GenBank: NM 003998]-left, 5'-TGC CAA CAG ATG GCC CAT AC, right, 5'-TGT TCT TTT CAC TAG AGG CAC CA-3', VEGF-[GenBank: NM 003376]-left, 5'-CGC AAG AAA TCC CGG TAT AA-3', right, 5'-AAA TGC TTT CTC CGC TCT GA-3' and PBGD-[GenBank: NM 000190]-left, 5'-AGC TAT GAA GGA TGG GCA AC-3', right, 5'-TTG TAT GCT ATC TGA GCC GTC TA-3'.

### Scratch Assay

The scratch assay is an *in vitro *assay used to determine the capacity of endothelial cells to migrate into a simulated wound administered by a single scratch with a pipette tip across the diameter of a confluent monolayer of CEC to remove cells from a roughly uniform area with in the well. Cells were grown to confluence in six well plates in ECM growth media. Prior to treatments, cells were placed into ECM containing 1% FBS. Cells were then exposed to 5 μM PI and/or 500 μM Vitamin E for 48 h. Cells undergoing Vitamin E treatment were pre-treated 1 h before, and throughout the PI treatment. Following scratching, media with or without treatments was replaced to remove floating cells and debris. The width and area of each scratch in each condition was recorded in 10 random and separate fields of view (phase contrast, 10×, Olympus, Melville, NY) and cells were digitally photographed using a MicroFire digital camera (Olympus, Melville, NY) and Optronics PictureFrame 2.1 imaging software (Optronics, Goleta, CA). Cells were incubated at 37°C in 5% CO_2_. Observations and photography were repeated 6 h later to monitor cell migration into the clearing formed by the scratch. Calculations of the area formed by the scratch at time 0 and 6 h were conducted using ImageJ 1.37v software (NIH, Bethesda, MD).

### Matrigel Angiogenesis Assay

The Matri-gel assay utilizes a soluble basement membrane preparation to provide a physiologically relevant environment where endothelial cells can migrate, branch and form ring-structures reminiscent of capillary tubules. Untreated control and CEC treated with NFV, Vitamin E, LY29004, Vitamin E/NFV or LY29004/NFV for 48 h were then cultured with treatments onto Growth Factor Reduced Matri-gel Matrix (BD Biosciences, Bedford, MA). After 6–8 h, the three-dimensional organization of the cells was examined microscopically (phase contrast, 10×, Olympus, Melville, NY) and digitally photographed using a MicroFire digital camera (Olympus) and Optronics PictureFrame 2.1 imaging software (Optronics, Goleta, CA). The average number of ring formations, cell extensions and extension lengths from 10 randomly selected fields of view from each treatment condition were photographed and compared for statistical significance.

### Statistical Analyses

Data were analyzed using Fischer's PLSD or one-way analysis of variance (ANOVA) with post hoc Dunnett's or Tukey-Kramer using Graph Pad Prism Software, (GraphPad Software Inc. San Diego, CA). All results are expressed as mean ± SEM, n ≥ 3.

## Results

### HIV PIs do not significantly affect cell viability in CEC

**Figure 1:**
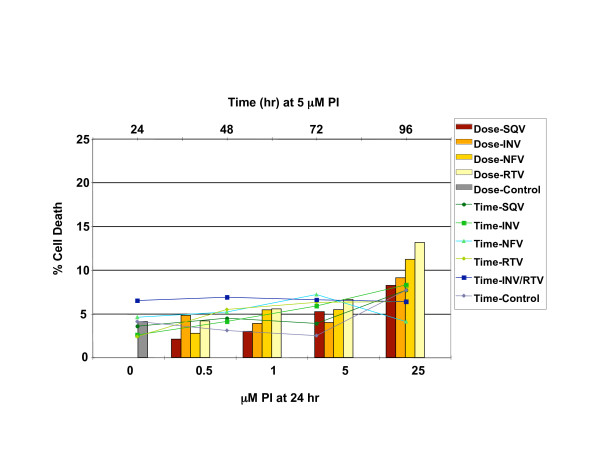
**HIV PIs do not affect cell viability in CEC**. Trypan blue staining for CEC viability was conducted after treatment with saquinavir (SQV), indinavir (INV), nelfinavir (NFV), or Ritonavir (RTV) for 24 h at 0, 0.5, 1, 5 and 25 μM dose concentrations. Trypan blue staining was conducted for CEC viability after treatment with 5 μM saquinavir (SQV), 5 μM indinavir (INV), 5 μM nelfinavir (NFV), 5 μM ritonavir (RTV), or 5 μM INV/1 μM RTV for 24, 48, 72 and 96 h. Percent cell death was calculated for each condition.

To determine whether exposure to reported plasma concentrations of HIV PIs affected CEC viability [19], dose and time course assays were performed. Treatment of CEC with 5 μM PIs up to 96 h resulted in less than 8% cell death compared to 4% for untreated controls (Figure 1). Thus, doses of 5 μM individual PI or 5 μM INV/1 μM RTV were used for all further experiments.

### HIV PIs induce the generation of ROS in CEC

**Figure 2:**
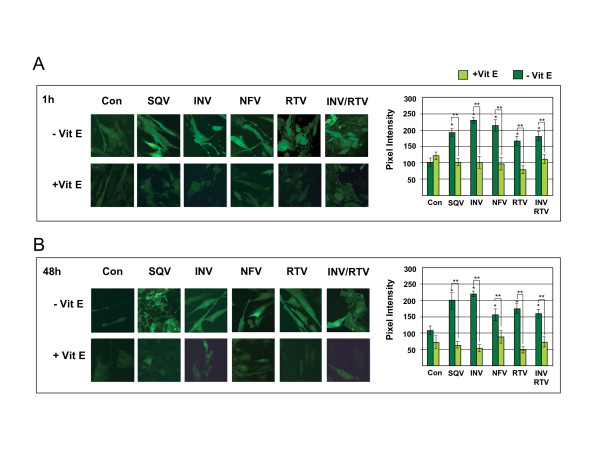
**Vitamin E prevents HIV PI-induced oxidative stress in CEC**. H_2_DCFDA staining was performed to determine the levels of ROS in cells treated with 5 μM saquinavir (SQV), indinavir (INV), nelfinavir (NFV), ritonavir (RTV), or 5 μM INV/1 μM RTV, with and without 1 h pre-treatment with Vitamin E to PI exposure for A) 1 h and B) 48 h. Pixel intensity from fluorescent H_2_DCFDA staining was measured by fluorescence confocal microscopy and graphed. Original magnification was 62×. For 1 h PI treatments, *p ≤ 0.05 by one way ANOVA with Dunnett's post hoc test when comparing to untreated control, and ** p ≤ 0.001 by Fisher's post hoc test when comparing PI treatment without Vitamin E to PIs with Vitamin E. For 48 h treatments, *p ≤ 0.05 by one way ANOVA with Dunnett's post hoc test when comparing to untreated control, and ** p ≤ 0.005 by Fisher's post hoc test when comparing PI treatment without Vitamin E to PI with Vitamin E. Graphs reflect results from three or more separate experiments.

Reported plasma concentrations of PIs do not cause significant CEC death *in vitro*, however studies show that PI exposure compromises cell fitness and signaling by inducing oxidative stress in non-cerebral endothelial cells [5,6]. Therefore, to determine whether PI exposure induced oxidative stress in CEC, ROS levels were measured by H_2_DCFDA staining in PI-treated cells. Treating CEC with 5 μM PIs or 5 μM INV/1 μM RTV resulted in significantly increased ROS after only 1 h (p ≤ 0.05, Figure 2A). Compared to untreated CEC, ROS levels in 1 h PI-treated CEC ranged from a 2.29-fold increase with INV treatment to 1.66-fold increase with RTV treatment. A similar range was observed in 48 h PI treated cells with a 2.03-fold increase with INV treatment to a 1.44-fold increase with NFV treatment (p ≤ 0.05) (Figure 2B). On the other hand, 1 h pre-treatment with the antioxidant, Vitamin E, prevented the generation of PI-induced ROS in both the 1 h and 48 h treated cells (p ≤ 0.001 and p ≤ 0.005, respectively) (Figure 2). These results show that the PIs tested induce significant levels of ROS in CEC, but Vitamin E pre-treatment prevents PI-induced ROS generation.

### PIs decrease Notch 4 protein expression in CEC

**Figure 3:**
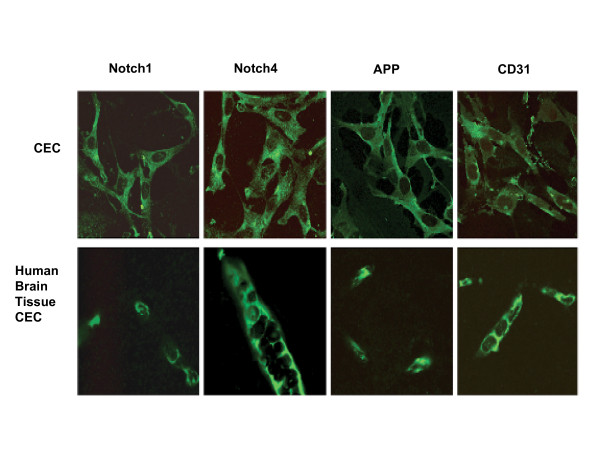
**Comparison of the expression of Notch 1, Notch 4, and APP in *in vitro *CEC and human brain frontal cortex**. Notch 1, Notch 4, and APP expression and localization were assessed by immunocytochemical labeling of cerebral endothelial cells (CEC) (top panels) and paraffin embedded human frontal cortex tissue (lower panels). CD31 was used as an endothelial cell-specific marker. Baseline immunoreactivity was observed by fluorescent confocal microscopy. Original magnification is 62×.

Because Notch is important to maintain CEC fitness and Notch signaling is affected by ROS [21], we first assessed baseline expression levels of the γ-secretase substrates Notch 1, Notch 4, and APP in CEC. For comparison to CEC, immunoreactivity of Notch 1, Notch 4, APP and CD31 was assessed in tissue sections from human brain frontal cortex. Immunological labeling showed that CEC express Notch 1, Notch 4, and APP in patterns comparable to those observed in the microvascular cells in human brain tissue (Figure 3).

**Figure 4:**
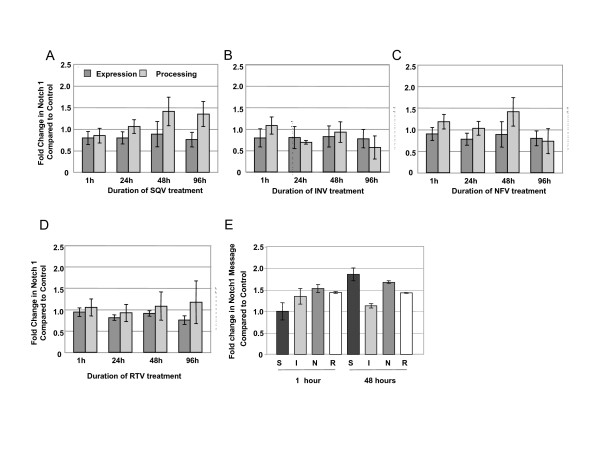
**HIV PIs do not affect Notch 1 expression or processing in CEC**. Western analyses of the expression of total Notch 1 (dark bars) and the percentage of Notch 1 processing (NICD) (lighter bars) after 5 μM exposure to A) saquinavir (SQV), B) indinavir (INV), C) nelfinavir (NFV), and D) ritonavir (RTV) for various lengths of time (1, 24, 48 and 96 h). E) quantitative real-time PCR analyses of Notch 1 mRNA levels after exposure to saquinavir (S), indinavir (I), nelfinavir (N), or ritonavir (R) for 1 or 48 h. No statistically significant differences were calculated by one way ANOVA. Graphs reflect results from three or more separate experiments.

**Figure 5:**
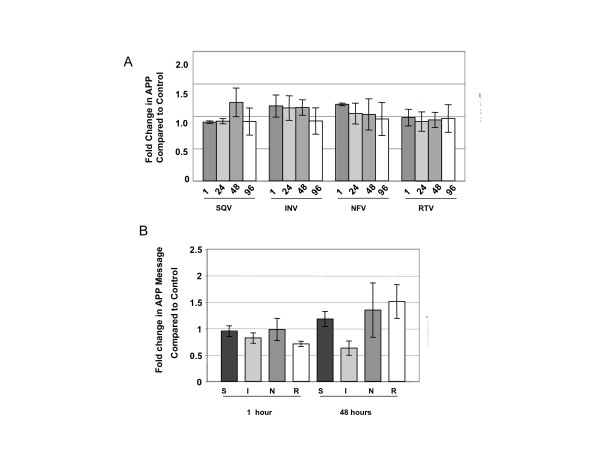
**HIV PIs do not affect APP expression in CEC**. A) Western analyses of the expression of total APP after 5 μM exposure to A) saquinavir (SQV), indinavir (INV), nelfinavir (NFV), and ritonavir (RTV) for various lengths of time (1, 24, 48 and 96 h). B) quantitative real-time PCR analyses of APP mRNA levels after exposure to saquinavir (S), indinavir (I), nelfinavir (N), or ritonavir (R) for 1 or 48 h. No statistically significant differences were calculated by one way ANOVA. Graphs reflect results from three or more separate experiments.

Exposure of CEC to PIs had no significant effects on total Notch 1 expression or processing which is the percent of total Notch represented by NICD (Figure 4A–D) or on Notch 1 messenger RNA levels (Figure 4E). Likewise, PIs had no significant effect on protein (Figure 5A) or messenger RNA levels (Figure 5B) of APP. We were unable to detect processed APP in CEC, thus, percent processed was not addressed.

**Figure 6:**
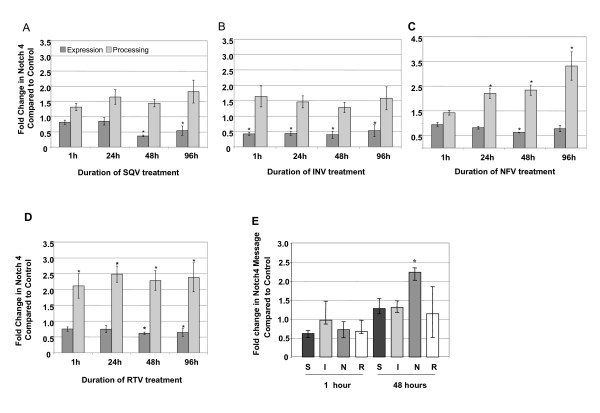
**HIV PIs affect Notch 4 protein expression and processing in CEC**. Western analyses of the expression of total Notch 4 (dark bars) (*p ≤ 0.002) and the percentage of Notch 4 processing (NICD) (lighter bars) after 5 μM exposure to A) saquinavir (SQV), B) indinavir (INV), C) nelfinavir (NFV) (*p ≤ 0.007), and D) ritonavir (RTV) (*p ≤ 0.001) for various lengths of time (1, 24, 48 and 96 h). E) quantitative real-time PCR analyses of Notch4 mRNA levels after exposure to saquinavir (S), indinavir (I), nelfinavir (N), or ritonavir (R) for 1 or 48 h. *p ≤ 0.001 by one way ANOVA with Dunnett's post hoc test when compared to untreated control. Graphs reflect results from three or more separate experiments.

On the other hand, SQV, INV, NFV and RTV significantly decreased total Notch 4 protein expression in CEC (p ≤ 0.002) (Figure 6A–D). Interestingly, even though total protein levels decreased, NFV and RTV *increased *the percentage of Notch 4 NICD. Through out the 96 h treatment course, exposure of CEC to NFV and RTV increased Notch 4 NICD levels significantly by ≥ 2 fold compared to control (p ≤ 0.007, p ≤ 0.001, respectively, Figure 6C and 6D). Only slight increases in Notch 4 NICD after SQV and INV treatment were observed, and increases did not reach statistical significance (Figure 6A and 6B). Importantly, since PI treatments decreased the levels of Notch 4 total protein, calculations were made to determine if the increase in Notch 4 NICD observed in the PI-treated cells exceeded the amount of NICD in control, untreated cells. Our results clearly show that levels of NICD in PI-treated cells are significantly higher than those in control cells relative to the amount of unprocessed (full length) Notch 4. Messenger RNA levels of Notch 4 were increased significantly by NFV after 48 h, whereas, other PIs had no significant effects (p < 0.001, Figure 6E).

**Figure 7:**
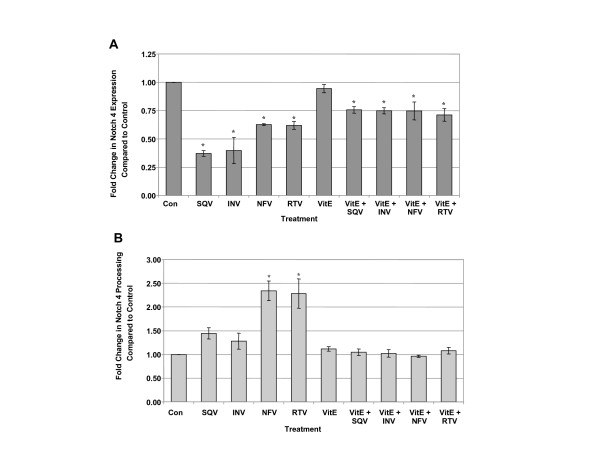
**Effects of Vitamin E on PI-induced changes in Notch 4 expression and processing**. Western analyses of CEC exposed to 5 μM saquinavir (SQV), indinavir (INV), nelfinavir (NFV), and ritonavir (RTV) for 48 h with or without Vitamin E pre-treatment (VitE). A) Expression levels of total Notch 4, B) percentage of total Notch 4 processed to NICD. * p ≤ 0.005 by one way ANOVA with Dunnett's post hoc test when compared to untreated control. Graphs reflect results from three separate experiments.

Since Vitamin E blocked ROS induced by PIs (Figure 1), and ROS is known to affect Notch expression and cleavage to NICD, CEC were exposed to SQV, INV, NFV or RTV for 48 h with or without Vitamin E pre-treatment to determine if Vitamin E could block the PI-induced alterations. Although Vitamin E pre-treatment prevented to some degree PI-induced decreases in Notch 4 total protein, levels remained significantly lower in PI-treated cells than in untreated controls (p ≤ 0.005, Figure 7A). On the other hand, Vitamin E blocked PI-induced increases in levels of Notch 4 NICD (p ≤ 0.005, Figure 7B). Exposure of CEC to other ROS inducers, cobalt chloride and H_2_O_2 _significantly increased levels of ROS as reported by other studies, but had no significant effects on Notch 4 expression or NICD levels (data not shown). These results suggest that induction of ROS by PIs may contribute to changes in Notch 4 expression and NICD levels. Since the percentage of Notch 4 that was detected as NICD in PI-treated cells was increased (Figure 4), immunocytochemical localization experiments using an antibody specific for processed Notch 4 were conducted to determine NICD cellular localization within PI-treated CEC.

### Nelfinavir exposure increases NICD nuclear localization in CEC

**Figure 8:**
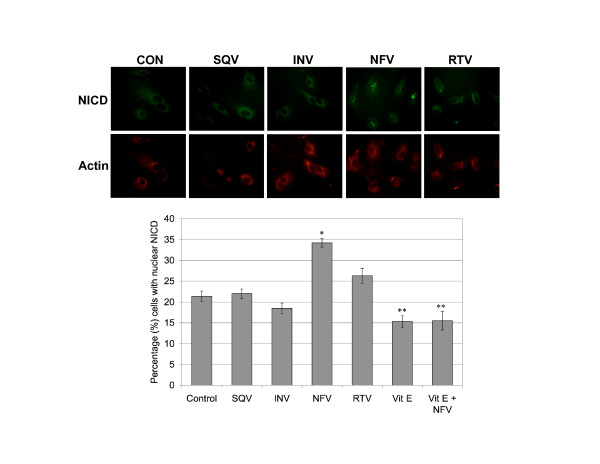
**Nelfinavir increases NICD nuclear localization**. CEC exposed to saquinavir (SQV), indinavir (INV), nelfinavir (NFV), and ritonavir (RTV) for 48 h were assessed for nuclear localization of NICD (green) and double labeled with actin (red). The percentage of cells positive for NICD nuclear localization was calculated by counting at least 10 random fields of view and at least 200 cells per condition. 60× magnification. *p ≤ 0.001 by one way ANOVA with Dunnett's post hoc tests when compared to control. **p ≤ 0.001 by one way ANOVA with Tukey-Kramer post hoc tests when compared to NFV treated cells.

CEC untreated, and treated with PIs and/or Vitamin E for 48 h were assessed for both cytosolic and nuclear NICD immunoreactivity. Nuclear localization of NICD was detected in 21% of untreated CEC, 22% with SQV, 19% with INV and 26% with RTV (Figure 8). However, NFV-treated CEC displayed the highest percentage (34%) of cells positive for NICD nuclear localization (p ≤ 0.001) (Figure 8B). On the other hand, Vitamin E treatment alone resulted in decreased NICD nuclear localization (15%), compared to control. As expected, Vitamin E pre-treatment blocked the dramatic increase in nuclear NICD caused by NFV, dropping the percent cells positive down to 15.5% (Figure 8B). These results indicate that the NFV-induced increases in levels of NICD (Figure 3C, D, 4B) are accompanied by increased NICD localization to the nucleus and that Vitamin E pre-treatment prevents NFV-induced increased nuclear localization of NICD (Figure 8B).

### NFV-induced changes in Notch 4 processing are independent of GSK-β phosphorylation levels

**Figure 9:**
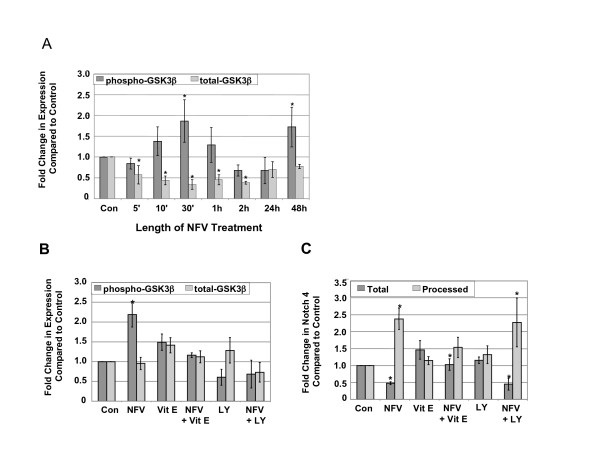
**NFV-induced changes in Notch 4 processing are independent of GSK3-β phosphorylation levels**. Western analyses of A) CEC exposed to NFV for varying lengths of time. Phosphorylated GSK3-β (dark bars) (*p ≤ 0.02) and total GSK3-β (lighter bars) (*p ≤ 0.002), B) phospho- and total GSK3-β in NFV-treated CEC in the presence of Vitamin E (VE) or the PI3K inhibitor, LY29004 (LY) and C) Total and Processed Notch 4 in NFV-treated CEC in the presence of Vitamin E (VE) or the PI3K inhibitor, LY29004 (LY), *p ≤ 0.05 by one way ANOVA with Dunnett's post hoc test when compared to untreated control. Graphs reflect results from three separate experiments.

Since GSK3-β phosphorylation levels are reported to be important in Notch signaling [18,22,23], the effects of NFV on GSK3-β phosphorylation levels were determined in CEC. Levels of phosphorylated GSK3-β followed a cyclic pattern over the NFV treatment time course, increasing significantly after 30 min and 48 h exposure to NFV (p ≤ 0.02) (Figure 9A). Total GSK3-β levels in NFV-treated CEC decreased significantly in early time points compared to control (p ≤ 0.002), but returned to control levels at 24 and 48 h (Figure 9A). Because, 48 h of NFV exposure resulted in significant increases in GSK3-β phosphorylation levels and in ROS generation (Figure 2B), we sought to determine the effects of GSK3-β phosphorylation on Notch 4 expression and processing, by treating CEC with NFV in the presence or absence of the PI3K inhibitor, LY29004 to prevent downstream AKT-mediated phosphorylation of GSK3-β. Treatment with the PI3K inhibitor blocked the increase in GSK3-β phosphorylation levels observed after NFV exposure, but had no effect on total GSK3-β (Figure 9B). Vitamin E, on the other hand, prevented changes in total and phospho-GSK3-β upon exposure to NFV, but alone had no effect on either total or phospho-GSK3-β (Figure 9B). However, blocking GSK3-β phosphorylation had no affect on Notch 4 expression or processing induced by NFV (Figure 9C). These results show that changes in Notch 4 processing upon exposure to NFV are independent of GSK3-β phosphorylation, but rather are related to NFV-induced ROS generation.

### NFV induces the expression of NICD regulated proteins

**Figure 10:**
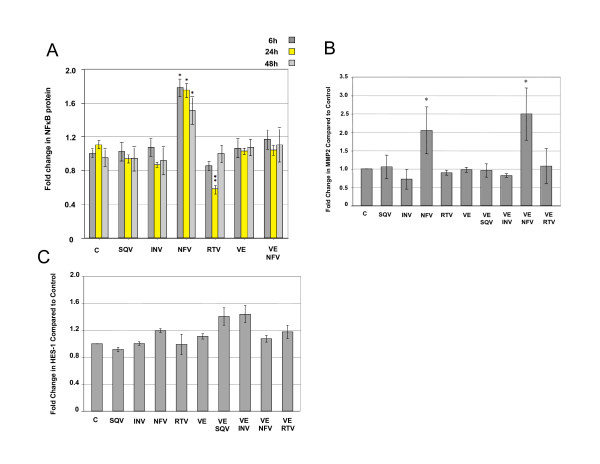
**NFV induces the expression of NICD gene targets in CEC**. Western analyses of A) NFκB (*p ≤ 0.005, ** p ≤ 0.05) after 6, 24 and 48 h exposure to 5 μM saquinavir (S), indinavir (I), nelfinavir (N), ritonavir (R), Vitamin E (VE), or VE/N, B) MMP2 (*p ≤ 0.01) and D) HES-1 protein levels after 48 h exposure to 5 μM saquinavir (S), indinavir (I), nelfinavir (N), ritonavir (R), with and without vitamin E (VE) pre-treatment. P values determined by one way ANOVA with Dunnett's post hoc tests when compared to control. Graphs reflect results from three or more separate experiments.

Genes regulated by NICD include, among others, the NFκB whose expression is induced by Notch [4,10,11]. Therefore, we assessed whether NFV affected the expression of NFκB, and other Notch targets [10,11]. NFκB protein expression was increased significantly (p < 0.01) after 6, 24 and 48 h NFV treatment, whereas, other PIs had no significant effects (Figure 10A). Vitamin E pre-treatment also blocked NFV-induced increases in NFκB. No changes were observed in NFκB messenger RNA after 1, 24 or 48 h NFV treatment (data not shown). After 48 h exposure to NFV significant increases were detected in MMP2 (p < 0.01, Figure 10B), an NICD target regulated by NFκB. MMP2 messenger RNA levels were increased after exposure to NFV (1 h, p < 0.01, Figure 10C), whereas, other PIs induced no changes in MMP2 messenger RNA. Although Vitamin E alone had no effect on NFκB and MMP2, pre-treatment with Vitamin E blocked NFV induced increases in (Figure 10A–C).

Increased VEGF protein was detected 1 h post-NFV treatment, but levels did not reach statistical significance (data not shown). Levels of VEGF protein returned to baseline after 24 and 48 h NFV exposure. No changes in VEGF messenger RNA levels were detected (data not shown). Neither message nor protein levels of HES-1 (Figure 10D) and HIF-1α were significantly affected by NFV (not shown).

The effects of NFV on Notch 4 expression and processing, NICD nuclear localization, and the expression of proteins related directly to Notch 4 signaling, are important for angiogenesis, thus CEC exposed to NFV were assayed for angiogenic capacity.

### NFV does not significantly hinder CEC angiogenic capacity

**Figure 11:**
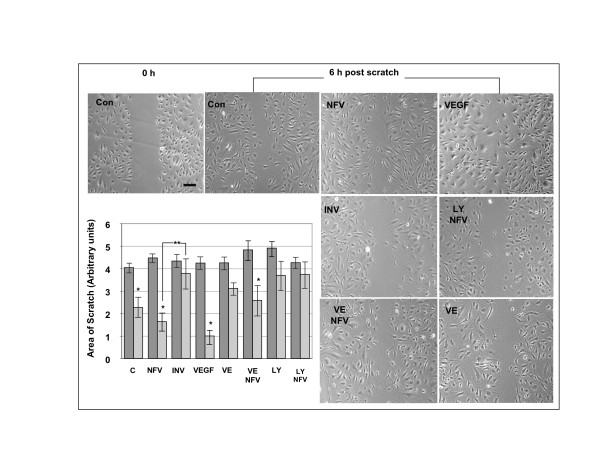
**Scratch assay showing that NFV does not interfere with CEC migration**. Panels show representative images of untreated CEC (C), or cells treated with Nelfinavir (NFV), Indinavir (INV), VEGF, vitamin E (VE) or LY29004 (LY). Top left panel (Con) shows a representative image of untreated CEC immediately post-scratch (0 h). All other panels show CEC 6 h post scratch. The graph represents results from measurements of the area of the scratch from at least 10 separate and random fields of view from three separate experiments. * p ≤ 0.017 by one way ANOVA with Dunnett's post hoc test when compared to untreated control. ** p < 0.001. Bar = μm

The scratch assay is an *in vitro *model for mechanical endothelial cell injury used to measure angiogenic capacity, proliferation and migration post-scratch. After 48 h treatments and scratch (0 h), CEC were allowed to recover for 6 h to assess capacity of cells to migrate to fill the area devoid of cells. After 6 h post-scratch recovery, untreated control cells (p < 0.001), cells treated with NFV (p = 0.017), and VEGF (p < 0.0001) showed significant decreases in the scratched area (Figure 11) indicating migration capacity. As expected, INV treatment inhibited cell migration into the denuded area. Vitamin E pre-treatment hindered the recovery of CEC treated with NFV, although levels of recovery in the Vitamin E/NFV CEC still reached significance (p < 0.01). Likewise, angiogenesis was blocked in cells treated with the inhibitor of GSK3-β phosphorylation with or without NFV (Figure 11), indicating that blocking GSK3-β phosphorylation inhibits angiogenesis in the presence or absence of NFV.

**Figure 12:**
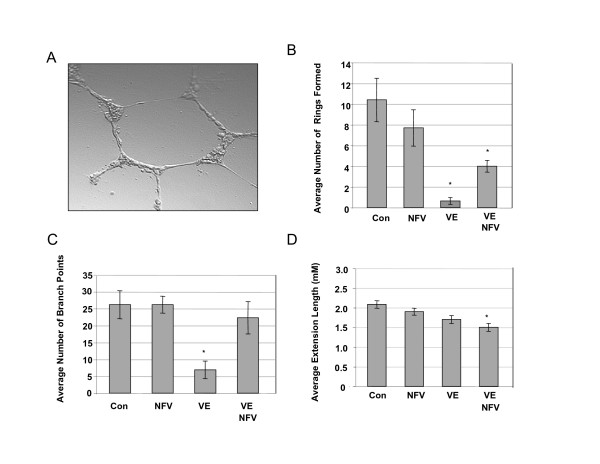
**NFV does not interfere with angiogenic capacity in CEC grown in a collagen matrix**. **A) Representative image of untreated CEC in matrigel forming aring structure and branching**. B-D) Results from Matrigel angiogenesis assay showing key elements of angiogenic behavior in CEC treated with Nelfinavir (NFV), vitamin E (VE), NFV/VE, or untreated control, B) Number of rings formed, *p ≤ 0.0001, C) Number of branch points, *p ≤ 0.0001, D) Extension lengths, *p ≤ 0.01. Results are from at least 10 separate fields of view in three separate experiments. P value was calculated by one way ANOVA with Dunnett's post hoc test when compared to untreated control.

To further characterize these findings, CEC were plated into reduced growth factor matri-gel matrix to measure three angiogenically relevant parameters (Figure 12A): ring formation, number of branches and extension lengths. Although fewer, the number of rings formed in NFV treated CEC were not significantly different from untreated control (Figure 12B). Vitamin E with (p < 0.0001) or without NFV (p < 0.001) exposure significantly blocked ring formation compared to control and NFV (Figure 9B). The numbers of branch points observed in NFV-treated CEC were not significantly different from control (Figure 9C); whereas, Vitamin E-treated CEC displayed significantly fewer branches than untreated or NFV treated (p < 0.0001, Figure 12C). Interestingly, in the presence of NFV, VE pre-treatment was unable to inhibit branching with numbers observed reflecting those in untreated or NFV-treated CEC (Figure 9C). The third parameter measured by the matri-gel assay, extension length indicated no significant differences between control and NFV, whereas, Vitamin E, and VE/NFV extensions were significantly shorter (p < 0.01) (Figure 9D). INV significantly blocked ring formation and branching, and shortened extension lengths in CEC (data not shown). Our results indicate that NFV does not affect negatively *in vitro *cell migration, ring formation or branching of CEC.

Together, the results from our study show that HIV-PIs induce ROS in CEC, but have no adverse affect on cell survival. PIs decrease total Notch 4 protein expression independently of ROS generation. All PIs tested increase Notch 4 processing with NFV and RTV inducing significant increases in NICD. Vitamin E pre-treatment blocks NFV-induced increases in NICD and phosphorylation of GSK3-β, but blocking GSK3-β phosphorylation has no effect on Notch 4 processing. It remains to be determined whether the effects of NFV on GSK3-β play a role in GSK3-β mediated NICD phosphorylation. SQV, NFV and RTV significantly increase the percentage of cells with NICD nuclear localization. NFV affects expression of NICD target genes and does not appear to inhibit significantly angiogenesis. Our results reflect previous findings that different PIs exert variable effects on cerebral endothelial cells, the consequences of which may be important during states of CNS inflammation during viral rebound or in HIV encephalitis. Given the importance of Notch 4 signaling in endothelial cell fitness and the crucial role of proper CEC functioning in maintaining blood brain barrier integrity, increased understanding of the interactions among antiretroviral medications and these signaling pathways is warranted.

## Discussion

Even though human protease enzymes are dissimilar from the HIV protease [26], inhibition of the human aspartic protease by HIV PIs is one of the many unpredicted effects reported [24-26]. In this regard, Notch is a substrate of the aspartyl protease, γ-secretase, that processes NICD for translocation to the nucleus. In the nucleus, NICD associates with the CSL family of DNA-binding proteins to form transcription activator complexes that regulate the expression of, among others, NFκB and VEGF [14,15]. Moreover, HIV PIs, particularly NFV, are implicated in disruption of the Notch pathway in the HIV-related neoplasm, Kaposi's sarcoma [7]. Recently, HIV protease inhibitors SQV and INV have been shown to induce regression of Kaposi's sarcoma [6,27]. Based on these findings, we originally predicted that Notch and APP would be affected by HIV-PIs in a similar manner. Likewise, previous studies show that the mechanisms by which the γ-secretase complex acts are common among these substrates [28], and our immunological data indicate substantial levels of APP, Notch 1 and Notch 4 proteins in CEC. Our results show however, that HIV PIs induce significant changes in only Notch 4. On the other hand, because Notch 4 is expressed predominantly by endothelial cells, [13,12], it stands to reason that this substrate would be affected most dramatically.

Importantly, all PIs tested in our study induced similar trends in the expression and processing of Notch 4, in that all PIs induced ROS, decreased total Notch 4 protein, and increased the percentage of Notch 4 that was processed to NICD. Likewise, the effects of Vitamin E on CEC treated with all PIs tested were similar. For example, Vitamin E blocked the production of ROS, and prevented the increased processing to NICD. Moreover, Vitamin E was able to block partially the decreases in total Notch 4 protein observed after PI treatment. The contribution of ROS to these changes may involve its capacity as an anti-oxidant sensitive second messenger in response to growth factor and cytokine stimuli [29]. In support of this possibility, both the Notch pathway and ROS-induced changes in GSK3-β signaling are linked tightly to endothelial cell angiogenic capacity [22,30-33].

Conversely, neither Vitamin E pre-treatment nor blocking GSK3-β phosphorylation significantly affected PI-induced decreases in total Notch 4 protein expression, suggesting that the overall decrease in total Notch 4 protein may occur by the many post-transcriptional modifications required prior to NICD processing and localization to the nucleus. Possible contributors may include the components of accessory proteins, ligands and proteases with which Notch 4 must interact prior to NICD release [34]. These processes include ubiquitination, endocytosis and conformational changes that permit cleavage of Notch to NICD [34,35].

The effects of PIs on Notch 4 in CEC follow similar trends, but the significance level of CEC response to PIs differs depending on the PI used. From our studies it appears that all PIs affect Notch 4, but that NFV induces the most significant changes in Notch 4 that follow through to cause increased NICD nuclear localization. Although numerous studies, including findings from our group, clearly show that different PIs have diverse effects on signaling in a given cell type [27,30-32], mechanisms for PI-specific effects are largely unknown. Furthermore, many studies showing that HIV-PIs alter signaling in non-cerebral endothelial cells [5,6,8,9] address the effects of only one or two PIs. Our findings show that all PIs effect Notch4 in a similar manner, but that NFV induces much more significant changes than the other PIs tested. On the other hand, studies addressing unintended effects of PIs on cell signaling show consistently that different PIs induce variable cellular responses [8,32,36].

Potential contributors to the differential effects of PIs on signaling in CEC may involve PI-specific substrate activation of the P-glycoprotein or multi-drug resistance protein since numerous studies show that PIs differ significantly in their substrate affinity and in their ability to activate these efflux transporters [37-39]. The involvement of this mechanism is attractive because both P-glycoprotein and multi-drug resistant protein are involved not only in efflux-dependent signaling but also in numerous pathways that contribute to diverse efflux-independent endothelial cell functions including caveolar-regulated intracellular trafficking and NFκB activation, cell survival, differentiation and proliferation [40]. In addition, the serum protein binding capacity among HIV PIs differs significantly and influences greatly their anti-viral activity and uptake into CEC [36,41].

Even though both NFV and RTV increased cellular NICD, only NFV increased NICD nuclear localization. Although the mechanism for this result is unclear, several potential contributors exist. For example, the degree of Notch ligand expression also can interfere, by an unknown mechanism, with NICD translocation to the nucleus [42]. In this regard, our preliminary data (not shown) thus far, show that HIV PIs do not affect the Notch 4 ligand, Delta 4, but studies are currently underway addressing potential ligand contributions. Another possible contributor to the observed PI-specific effects on Notch 4 may involve regulation of components of the transcription factor complex of CSL DNA-binding proteins. NICD has no intrinsic DNA binding activity without physical binding to CSL family of DNA-binding proteins [43] thus, NFV may promote NICD localization indirectly by affecting one or more components of the transcription factor complex. Studies by Pore et al., report that in response to hypoxia, NFV induces HIF-1α, decreases VEGF and inhibits angiogenesis in glioblastoma cells via the AKT pathway, but has no effect on normal astrocytes [31] showing that the effects of NFV are dependent on cell type and on other factors such as oxygenation levels. Results from our study clearly show that Notch expression, processing, localization and signaling are affected significantly by exposure to certain HIV PIs by mechanisms involving ROS generation. Thus, further investigation into the membrane bound forms of Notch and its association with accessory proteins involved in proteolysis during cleavage is warranted. Such studies will help to elucidate the point in the Notch 4 signaling cascade that is affected by HIV PIs.

It is well established that some PIs such as INV and SQV inhibit angiogenesis as illustrated in an HIV-related Kaposi sarcoma mouse model [6], however the potency and effects of different PIs on endothelial cell functioning are quite variable [44]. In agreement with previous studies, our results showed that INV inhibited significantly angiogenesis in parallel scratch and matri-gel assays. Pre-treatment with Vitamin E also inhibited significantly CEC migration into the wound area and blocked partially migration of CEC exposed to NFV. Interestingly, in the presence of both Vitamin E and NFV, ring formation and the number of branch points observed increased from Vitamin E treated cells alone. Thus, as reported by Navarra et al., Vitamin E does not alter angiogenesis in the presence of angiogenic stimuli; however, results of our study suggest that NFV may induce signaling in CEC sufficient to allow some aspects of angiogenesis even in the presence of Vitamin E [45]. Another recent study reported that a combination of radiation and NFV in a xenograft mouse model increases time to tumor re-growth compared to radiation alone. However, NFV alone had little effect on tumorogenesis [7,30]. Together these results suggest that NFV affects differently specific aspects of angiogenesis such as migration, ring formation, and branching. Thus, our results support the finding that NFV has effects on angiogenesis different from other PIs tested.

## Conclusion

Recent studies have shown that among the HIV PIs prescribed currently, NFV- and INV-containing HAART regimens significantly increased ROS and leukocyte recruitment [5] that in turn increases the likelihood of viral entry across the BBB into the CNS. However, NFV but not INV, has been linked to both clinical findings of increased incidence of cardiovascular [46] and endothelial cell dysfunction [5]. Moreover, Notch 4-mediated signaling plays a major role in endothelial cell growth, differentiation and angiogenesis [11]. Notch signaling is important in maintaining angiogenic properties of endothelial cells [11,47], and studies have shown that Notch in human neuroblastoma and neuronal cells is increased during conditions of increased ROS generation [48,49].

Although questions remain as to the specific mechanisms responsible for decreased Notch 4 protein upon exposure to PIs and NFV-specific increases in NICD nuclear localization, NFκB and MMP2 expression, our results showing that NFV effects significantly Notch 4 expression, processing and NICD localization in CEC are important to better understand potential signaling alterations among components of the cerebrovascular unit in HIV patients.

## Authors' contributions

TDL conceived and planned the experiments, AG, RH, YC and CP conducted all experiments and analyzed the data, TDL and RH wrote the manuscript. All authors read and approved the final manuscript.
